# Association of neurostructural biomarkers with secondary attention-deficit/hyperactivity disorder (ADHD) symptom severity in children with traumatic brain injury: a prospective cohort study

**DOI:** 10.1017/S0033291722002598

**Published:** 2023-08

**Authors:** Nicholas P. Ryan, Cathy Catroppa, Storm Courtney Ward, Keith Owen Yeates, Louise Crossley, Marisa Hollenkamp, Stephen Hearps, Miriam H. Beauchamp, Vicki A. Anderson

**Affiliations:** 1 Cognitive Neuroscience Unit, Deakin University, Geelong, Australia; 2 Clinical Sciences, Murdoch Children's Research Institute, Melbourne, Australia; 3 Department of Paediatrics, University of Melbourne, Australia; 4 Department of Psychology, Hotchkiss Brain Institute, and Alberta Children's Hospital Research Institute, The University of Calgary, Calgary, Alberta, Canada; 5 Department of Psychology, University of Montreal, Montreal, Canada; 6 Ste-Justine Research Center, Montreal, Quebec, Canada

**Keywords:** Attention-deficit/hyperactivity disorder, brain injuries, childhood, structural MRI

## Abstract

**Background:**

Despite a well-established link between childhood traumatic brain injury (TBI) and elevated secondary attention-deficit/hyperactivity disorder (s-ADHD) symptomology, the neurostructural correlates of these symptoms are largely unknown. Based on the influential ‘triple-network model’ of ADHD, this prospective longitudinal investigation aimed to (i) assess the effect of childhood TBI on brain morphometry of higher-order cognitive networks proposed to play a key role in ADHD pathophysiology, including the default-mode network (DMN), salience network (SN) and central executive network (CEN); and (ii) assess the independent prognostic value of DMN, SN and CEN morphometry in predicting s-ADHD symptom severity after childhood TBI.

**Methods:**

The study sample comprised 155 participants, including 112 children with medically confirmed mild-severe TBI ascertained from consecutive hospital admissions, and 43 typically developing (TD) children matched for age, sex and socio-economic status. High-resolution structural brain magnetic resonance imaging (MRI) sequences were acquired sub-acutely in a subset of 103 children with TBI and 34 TD children. Parents completed well-validated measures of ADHD symptom severity at 12-months post injury.

**Results:**

Relative to TD children and those with milder levels of TBI severity (mild, complicated mild, moderate), children with severe TBI showed altered brain morphometry within large-scale, higher-order cognitive networks, including significantly diminished grey matter volumes within the DMN, SN and CEN. When compared with the TD group, the TBI group showed significantly higher ADHD symptomatology and higher rates of clinically elevated symptoms. In multivariable models adjusted for other well-established risk factors, altered DMN morphometry independently predicted higher s-ADHD symptomatology at 12-months post-injury, whilst SN and CEN morphometry were not significant independent predictors.

**Conclusions:**

Our prospective study findings suggest that neurostructural alterations within higher-order cognitive circuitry may represent a prospective risk factor for s-ADHD symptomatology at 12-months post-injury in children with TBI. High-resolution structural brain MRI has potential to provide early prognostic biomarkers that may help early identification of high-risk children with TBI who are likely to benefit from early surveillance and preventive measures to optimise long-term neuropsychiatric outcomes.

## Introduction

Traumatic brain injury (TBI) is a leading cause of childhood death and acquired disability worldwide (Dewan, Mummareddy, Wellons, & Bonfield, 2016). Childhood TBI has been linked to a range of debilitating neurobehavioral sequelae, including an increased risk for developing novel psychiatric disorders (Emery et al., 2016; Li & Liu, 2013). Attention-deficit/hyperactivity disorder (ADHD), defined by developmentally inappropriate and functionally impairing symptoms of inattention and/or hyperactivity/impulsivity (American Psychiatric Association, 2013), is the most frequent novel psychiatric disorder after childhood TBI with a prevalence of approximately 20% (Max et al., 2005a, 2005b, 2012; Narad et al., 2018). New-onset ADHD symptomatology occurring after TBI is referred to as *secondary ADHD* (s-ADHD) (Narad et al., 2018). Despite evidence that children with s-ADHD show significantly more functional difficulties than those without s-ADHD, risk factors for elevated s-ADHD symptomatology are not well understood (Gerring et al., 1998; Narad et al., 2018).

Prior childhood TBI research suggests that the substantial heterogeneity in s-ADHD symptom severity is at least partly explained by acute TBI severity. In a prospective cohort of 81 children with complicated-mild to severe TBI, Narad et al. (2018) found that children of all TBI severity levels had almost twice the risk for s-ADHD than age-matched orthopaedically injured children. The strongest associations were observed in children with severe TBI, who demonstrated a fourfold increased risk for developing s-ADHD (Narad et al., 2018). Similarly, a recent meta-analysis found that in children with severe TBI, the odds for ADHD were significantly higher than in children with other injuries (Asarnow, Newman, Weiss, & Su, 2021).

In addition to the role of acute TBI severity, evidence links higher s-ADHD symptomatology to non-injury-related risk factors, including poorer pre-injury child adaptive functioning (Max et al., 2005a, 2005b), lower family socio-economic status (SES) (Gerring et al., 1998; Max et al., 2005a, 2005b) and pre- and post-injury family dysfunction (Max et al., 1998; Narad et al., 2018). Interestingly, Max et al. (1998) found that when the effects of acute TBI severity, family psychiatric history, SES and pre-injury family functioning were modelled simultaneously, only poorer family functioning independently predicted higher ADHD symptomatology. While these findings suggest that pre-injury child and family environmental factors may interact with injury characteristics to heighten risk for s-ADHD symptomatology after childhood TBI (Gerring & Wade, 2012), the neurostructural brain correlates of these post-injury symptoms are largely unknown.

Based on the influential triple-network (TN) model of ADHD (Cai, Chen, Szegletes, Supekar, & Menon, 2018; Castellanos & Proal, 2012; Menon, 2011), one possibility is that s-ADHD symptomatology is associated with injury to large-scale, higher-order cognitive networks. According to the TN model, ADHD stems from aberrant structural and functional connectivity among three large-scale brain networks that function in concert to support top-down mental processes (Cai, Ryali, Chen, Li, & Menon, 2014): the central executive network (CEN), the default mode network (DMN) and the salience network (SN). The CEN, which is anchored in the dorsolateral prefrontal cortex and the posterior parietal cortex, plays a central role in mediating aspects of top-down cognitive control, including working memory, selective attention, set-shifting and inhibition (Owen, McMillan, Laird, & Bullmore, 2005; Wager & Smith, 2003). The DMN, which is anchored in the posterior cingulate cortex and the medial prefrontal cortex, is anti-correlated with the CEN and plays an indirect yet important role in higher-order cognitive processes. For instance, higher-order cognition relies on the availability of neuronal resources, which is accomplished by diminishing activation of the DMN during top-down mental processing (Baum et al., 2017; Gusnard, Akbudak, Shulman, & Raichle, 2001; Satterthwaite et al., 2013). The TN model of higher-order cognition also posits a central role for the SN, which is anchored in the anterior insula and the anterior cingulate cortex and is responsible for modulating the activation of the CEN and deactivation of the DMN during tasks requiring top-down cognitive processes (Cai et al., 2016; Sridharan, Levitin, & Menon, 2008).

The TN model of ADHD is supported by extensive meta-analytic evidence showing abnormal activation of the SN, CEN and DMN in participants with ADHD relative to neurotypical controls (Cortese et al., 2012). In one study, Cai et al. (2018) found that childhood ADHD is associated with significantly lower time-varying engagement of the SN with the CEN and DMN relative to typically developing (TD) children. These intermittently weaker cross-network interactions between the SN, CEN and DMN were also shown to correlate with ADHD symptom severity in two independent child ADHD cohorts. Consistent with these findings, analyses of structural MRI using voxel-based morphometry in child and adolescent samples have consistently linked ADHD to grey matter structural alterations in hub regions of the CEN, SN and DMN (Francx et al., 2016; Su et al., 2021). These findings suggest that ADHD may stem from aberrancies in structure and function of higher-order cognitive networks that are also highly susceptible to disruption in childhood TBI. However, no study has employed high-resolution structural brain MRI to establish whether elevated s-ADHD symptomatology after childhood TBI may be associated with neurostructural alterations within higher-order cognitive circuitry.

Several lines of behavioural and neuroimaging evidence suggest that childhood TBI is associated with abnormalities of large-scale, higher-order cognitive systems. At the group level, children with TBI demonstrate widespread impairments in top-down cognitive control, including poorer performance on tasks of cognitive flexibility, inhibition and working memory (Robinson et al., 2014). Consistent with these behavioural findings, moderate-severe childhood TBI is associated with aberrant functional connectivity within the DMN and CEN (Risen, Barber, Mostofsky, & Suskauer, 2015; Stephens et al., 2018), as well as chronic grey matter structural alterations within the CEN, SN and DMN (Dennis et al., 2013). While these findings suggest that higher-order cognitive circuitry may be vulnerable in childhood TBI, large prospective studies are needed to evaluate whether regional brain morphometry within these large-scale cognitive networks has prognostic value for predicting s-ADHD symptom severity in children with TBI.

The current, prospective investigation aimed to (i) assess the effect of childhood TBI on sub-acute brain morphometry of the DMN, SN and CEN, and ADHD symptom severity, compared to a TD comparison group; and (ii) evaluate the independent prognostic value of DMN, SN and CEN morphometry for prospectively predicting s-ADHD symptom severity after adjusting for other established risk factors for s-ADHD, including acute TBI severity, SES, family function and pre-injury child adaptive functioning.

We expected significant group differences in DMN, SN and CEN morphometry, such that the severe TBI group would show diminished DMN, SN and CEN volumes compared to TD controls and children with milder head injuries. Based on prior research (Narad et al., 2018), we hypothesised that the TBI group would display significantly higher ADHD symptomatology and significantly higher rates of borderline-clinical scores (age-adjusted *T* > 65) than the TD group. Finally, we expected that, after adjusting for other well-established risk factors, altered morphometry of the DMN, CEN and SN would prospectively predict higher s-ADHD symptomatology at 12-months post-injury.

## Methods

### Participants and procedure

Children with mild-severe TBI were prospectively recruited into a larger longitudinal study (Anderson et al., 2017), and represented consecutive admissions to the emergency department and intensive care unit at a state-wide paediatric level I trauma centre in Victoria, Australia. Age and sex-matched TD children were recruited from local community schools representing a range of socio-economic backgrounds. For the TBI group, we applied the following inclusion criteria: (i) 5–16 years of age at recruitment; and (ii) medically confirmed TBI, including availability of medical records to reliably determine TBI severity [i.e. Glasgow Coma Score (GCS), neurological findings]. Exclusion criteria were as follows: (i) suspected non-accidental injury or previous TBI; (ii) pre-injury history of congenital, neurological or neurodevelopmental diagnosis including ADHD, autism spectrum disorder and specific learning disorder, which were assessed using a parent interview-form administered upon initial study enrolment; and (iii) non-English speaking background. The human data reported in this study were obtained in compliance with the hospital Human Research Ethics committee. Parent consent and child assent were obtained prior to data collection.

In total, 112 TBI participants were enrolled in the study and were categorised as follows: (i) *mild TBI* (*n* = 57): GCS 13–15 on hospital admission, loss of consciousness (LOC) <1 h, negative brain CT/clinical MRI; (ii) *complicated-mild TBI* (*n* = 14): GCS 13–15 on admission, LOC <1 h, positive brain CT/clinical MRI finding; (iii) *moderate TBI* (*n* = 26): GCS 9–12 on hospital presentation, LOC 1–24 h; (iv) *severe TBI* (*n* = 15): GCS 3–8 on admission, LOC >24 h. The TD group (*n* = 43) were aged 5–16 years at recruitment and were group-matched to the TBI group on sex and SES. SES was assessed using the Australian Socioeconomic Index 2006 (McMillan, Beavis, & Jones, 2009), which transforms the Australian and New Zealand Standard Classification of Occupations (ANZSCO) codes into a continuous measure of occupational status ranging from 0 (labourers) through to 100 (medical practitioners).

As reported in Table 1, groups did not significantly differ on age, sex or SES. Since group differences for SES neared statistical significance (*p* = 0.050), we covaried for SES in all subsequent analyses. With respect to injury-related characteristics (Table 2), falls/blows were most frequent in children with mild, complicated-mild and moderate TBI. Motor vehicle crashes and falls/blows were equally common in children with severe TBI. Children with moderate and severe TBI were more likely to receive surgical intervention.

**Table 1.** Baseline and demographic characteristics of enrolled sample

	TD control	Mild TBI	Complicated-mild TBI	Moderate TBI	Severe TBI	*F/*χ^2^	*p*
*n* (participants)	43	57	14	26	15	–	–
Male, *n* (*%*)	24 (55.81)	44 (77.19)	8 (57.14)	16 (61.54)	8 (53.33)	6.68	0.154
Socio-economic status, *M* (s.d.)	76.75 (14.19)	70.17 (19.64)	58.61 (27.09)	60.88 (24.76)	70.66 (25.09)	2.43	0.050
Child IQ (WASI-II), *M* (s.d.)	104.35 (13.39)	100.10 (13.78)	96.57 (12.93)	94.96 (14.26)	94.33 (13.84)	3.89	0.091
Recruitment age (years), *M* (s.d.)	10.25 (3.04)	10.67 (2.36)	9.47 (2.44)	10.33 (2.49)	9.72 (3.01)	0.80	0.528
MRI age (years), *M* (s.d.)	10.41 (2.76)	10.80 (2.33)	9.57 (2.43)	10.37 (2.58)	10.41 (3.10)	0.66	0.621
Pre-injury baseline function, M (s.d.)							
Adaptive Behaviour Assessment System (ABAS-II): Social scale	10.44 (2.74)	10.02 (3.37)	9.46 (3.20)	10.00 (2.08)	9.86 (3.42)	0.32	0.862
ABAS-II Adaptive Composite	97.37 (15.38)	97.26 (17.15)	95.77 (16.15)	98.32 (13.08)	94.71 (15.75)	0.14	0.965
CBCL Internalizing Total	47.16 (8.31)	48.26 (11.25)	51.54 (12.33)	47.88 (9.58)	49.21 (9.58)	0.50	0.733
CBCL Externalizing Total	46.63 (6.99)	48.13 (10.09)	51.39 (10.40)	48.04 (11.23)	50.50 (9.88)	0.86	0.488

WASI-II, Wechsler Abbreviated Scale of Intelligence – Second Edition (WASI-II).

**Table 2.** Sample injury-related characteristics

	TD control	Mild TBI	Complicated-mild TBI	Moderate TBI	Severe TBI	*F/*χ^2^	*p*
Age at injury (yrs), *M* (s.d.)	–	10.67 (2.36)	9.47 (2.44)	10.33 (2.49)	9.72 (3.01)	1.20	0.312
Lowest GCS, *M* (s.d.)	–	14.54 (1.04)	13.93 (1.14)	11.50 (1.98)^a,b^	5.60 (2.20)^c,d,e^	148.99	*p* < 0.001
Hospital stay (days), *M* (s.d.)	–	0.48 (0.73)	2.30 (1.82)	4.75 (4.59)^a,b^	17.7 (13.9)^c,d,e^	38.83	*p* < 0.001
Surgical intervention, *n* (*%*)	–	0 (0)	0 (0)	8 (31)	7 (47)	32.07	*p* < 0.001
Cause of injury, n (%)						33.60	0.001
Motor vehicle crash (MVC) (car)	–	1 (2)	1 (7)	5 (19)	4 (27)	–	–
MVC (pedestrian/bike)	–	3 (5)	1 (7)	5 (19)	5 (33)	–	–
Fall (stationary)	–	13 (23)	4 (29)	8 (31)	2 (13)	–	–
Fall (moving)	–	22 (39)	7 (50)	7 (27)	3 (20)	–	–
Kicked/struck by object	–	18 (32)	1 (7)	1 (4)	1 (7)	–	–
MRI pathology, *n* (%)							
Frontal	–	–	4 (29)	14 (54)	10 (67)	27.46	*p* < 0.001
Extra-frontal	–	–	4 (29)	9 (35)	6 (40)	20.56	*p* < 0.001
Sub-cortical	–	–	1 (7)	4 (15)	4 (27)	16.27	0.003
Global volumes, cm^3^ (s.d.)							
Total brain volume	1232 (118)	1262 (116)	1204 (86)	1222 (113)	1175 (103)	1.752	0.142
Estimated total intracranial volume	1502 (138)	1536 (144)	1455 (119)	1500 (121)	1485 (180)	0.731	0.573
Grey matter total	755 (71)	779 (65)	748 (44)	750 (88)	696 (59)^c,e^	3.929	0.005
White matter total	435 (49.91)	439 (52.54)	415 (45)	433 (37)	427 (74)	0.349	0.845

GCS, Glasgow Coma Score.

*Note*: Statistically significant Bonferroni-corrected post-hoc analyses comparing ^a^mild TBI *v.* moderate TBI, ^b^complicated-mild TBI *v.* moderate TBI, ^c^mild TBI *v.* severe TBI, ^d^complicated-mild TBI *v.* severe TBI, ^e^moderate TBI *v.* severe TBI.

### Pre-injury functioning

The primary caregiver retrospectively evaluated the child's pre-injury functioning using the Child Behaviour Check List (CBCL-6–18) (Achenbach & Rescorla, 2001) and the Adaptive Behaviour Assessment System-II (ABAS-II) (Harrison & Oakland, 2003). The ABAS-II is a well-validated parent-rated measure of conceptual, practical and social adaptive skills, ratings of which are used to derive the Global Adaptive Composite (GAC; *M* = 100, s.d. = 15) reported in Table 1. No significant group differences were identified when the pre-injury adaptive function of the TBI groups was compared to adaptive function ratings of the TD group collected at time of recruitment (Table 1). Similarly, the TBI groups did not significantly differ from the TDC group on pre-injury externalizing symptoms or pre-injury internalizing symptoms on the CBCL-6–18 (Table 1).

### ADHD symptom severity at 12-months post-injury

CBCL-6–18 (Achenbach & Rescorla, 2001) is a valid and reliable parent-rated questionnaire designed to assess child emotional and behavioural problems. Age-adjusted *T* scores for the DSM-oriented ADHD Problems scale and the Attention Problems scale (*M* = 50; s.d. = 10) were used to assess ADHD symptom severity. The ADHD Problems subscale consists of seven items aligned with DSM criteria for ADHD and shares five items with the Attention Problems scale (10 items), which is designed to more comprehensively assess symptoms of inattention associated with ADHD. *T* scores of 65 and above were used to denote borderline-clinical range symptomatology.

### Family functioning at 6-months post-injury

The McMaster Family Assessment Device (FAD) – Global Functioning Scale (Epstein, Baldwin, & Bishop, 1983) is a well-validated parent-report questionnaire, with higher scores indicative of worse family functioning.

### High-resolution structural MRI

#### Image acquisition and pre-processing

Children were examined using high-resolution structural brain MRI acquired at 6 weeks post-injury (*M* = 42; s.d. = 29 days post-injury). As reported previously (Ryan et al., 2021), MR images were acquired on a 3 Tesla Siemens Trio scanner (Siemens Medical Systems, Erlangen, Germany) using a 32-Channel matrix head coil. In accordance with the protocol recommended by the National Institute of Neurological Disorders and Stroke Common Data Elements group, the MRI sequences included T1- and T2-weighted, axial T2-weighted fluid attenuated inversion recovery, axial susceptibility weighted and axial diffusion-weighted sequences. Quality control involved ranking the quality of T1-weighted images taking into account ringing, motion and susceptibility.

Of the 155 participants who underwent the research MR protocol, seven TBI participants and nine TD children were excluded due to motion artefact, dental artefact and/or incomplete data due to non-compliance. In addition, another two children with severe TBI had pronounced localised regions of encephalomalacia. In both cases, parenchymal distortion prohibited automated image analysis because of an absence of defining boundaries for image parcellation.

#### Morphometric analysis

Useable data for 137 participants were available for analysis. Cortical reconstruction and volumetric segmentation were completed using FreeSurfer Version 5.3 (http://surfer.nmr.mgh.harvard.edu.au). Details of the procedures are provided in previous publications (Fischl et al., 2004; Jovicich et al., 2009). Briefly, the processing includes the removal of non-brain tissue using a hybrid watershed/surface deformation procedure, automated Talairach transformation, segmentation of the subcortical WM and deep GM volumetric structures (Fischl et al., 2002), intensity normalisation (Sled, Zijdenbos, & Evans, 1998), tessellation of the GM–WM boundary, automated topology correction (Ségonne, Pacheco, & Fischl, 2007) and surface deformation following intensity gradients to optimally place the GM/WM and GM/CSF borders at the location where the greatest shift in intensity defines the transition to the other tissue class (Fischl & Dale, 2000). The resulting cortical models were registered to a spherical atlas, utilizing individual cortical folding patterns to match cortical geometry across subjects (Fischl, Sereno, Tootell, & Dale, 1999).

The cerebral cortex was parcellated into regions based on gyral and sulcal structure (Desikan et al., 2006). Results for each subject were visually inspected to ensure accuracy of registration, skull stripping, segmentation and cortical surface reconstruction. Manual editing, where necessary, was performed to optimise accuracy. The surface inaccuracies involving skull stripping or frank exclusion of brain parenchyma were edited either by (1) adding control points to aid FreeSurfer in the identification of white matter (since it uses the WM/GM boundary as a starting place for reconstructing the pial surface) (three cases) or (2) by fixing the skull strip by removing remaining dura (four cases).

Regions of interest (ROIs) defined in FreeSurfer variables (Desikan et al., 2006) were obtained by extracting volumes for each ROI bilaterally. Using the methodology outlined in previous childhood TBI research (Dennis et al., 2013; Hoskinson et al., 2019), composite measures of DMN, CEN and SN morphometry were derived by summing together the bilateral ROIs within each of the respective neural networks of interest – DMN, CEN and SN (Dennis et al., 2013; Hoskinson et al., 2019). Replicating the methodology from prior childhood TBI research (Dennis et al., 2013), the DMN composite was derived by summing the volumes of the bilateral ventromedial prefrontal cortex, posterior cingulate cortex, inferior parietal lobule and hippocampal formation. The CEN composite includes the dorsolateral prefrontal cortex, posterior parietal cortex, caudate nucleus and thalamus, whereas the SN composite includes the ventrolateral prefrontal cortex, insula, anterior cingulate cortex and amygdala. This process yielded three network-based morphometry variables (i.e. brain morphometry values for the DMN, CEN and SN, respectively) for use in subsequent analyses.

### Statistical analysis

SPSS Version 25.00 (IBM Corporation) was used for screening and analysis of all study data. Statistical assumptions were met for each analysis, unless otherwise stated. The variance inflation factor (VIF) was used to assess for multi-collinearity. One-way analysis of covariance was used to assess for group differences in ADHD symptom severity after adjustment for SES, participant sex and age. Significant omnibus results were followed by Bonferroni-corrected *post hoc* pairwise comparisons. Network-based morphometry was examined using multivariate analysis of covariance (MANCOVA). In these analyses, group membership was entered as the between-subjects factor and network-based morphometry (DMN *v.* CEN *v.* SN) was entered as the within-subjects factor.

Multivariable regression analyses were used to assess prospective relationships between network-based morphometry (DMN, SN, CEN) and ADHD symptom severity in children with TBI. In each multivariate model, measures of network-based morphometry were modelled simultaneously with adjustment for other established s-ADHD risk factors, including acute TBI severity indexed by the GCS, SES, post-injury family functioning and retrospective ratings of pre-injury child adaptive skills. Participant age, sex, pre-injury CBCL externalizing symptoms and estimated total intracranial volume (TIV) were also entered as covariates in all multivariate regression models. This approach enabled us to assess if measures of network-based brain morphometry are unique and therefore useful markers of s-ADHD symptom severity in children with TBI.

## Results

### Effect of childhood TBI on network-based brain morphometry

In total, 137 participants had useable MRI for FreeSurfer to produce volumetric data. A sensitivity analysis found no significant differences in the pre-injury, demographic or injury-related characteristics of those with and without useable MRI. Groups did not significantly differ on measures of estimated TIV or whole brain volume (Table 2). The time interval between day-of-injury and research MRI was not significantly associated with measures of global brain volume or network-based morphometry (all *p* ⩾ 0.25).

Group means and standard deviations for network-based morphometry are presented in Table 3. To complement these data, online Supplementary Figure S1 includes violin plots depicting network-based brain morphometry (i.e. DMN, CEN, SN volume) as a function of TBI severity group membership. As expected, the overall MANCOVA was statistically significant after adjusting for participant sex, age at MRI, TIV and SES [*F*_(5,129)_ = 8.97, *p* < 0.001, *η*^2^ = 0.218]. As shown in Table 3, significant overall group differences were documented for DMN, CEN and SN morphometry. Post-hoc pairwise comparisons (see superscripts in Table 3) indicated that, compared to the TD group and all other TBI severity groups (mild, complicated mild, moderate groups), children with severe TBI had diminished DMN, CEN and SN volumes.

**Table 3.** Group differences in DMN, SN and CEN morphometry and ADHD symptom severity

	TD control	Mild TBI	Complicated-mild TBI	Moderate TBI	Severe TBI	*F*	*p*	*η*^2^
Morphometry cm^3^ (s.d.)								
DMN	116.50 (10.89)	118.68 (10.20)	114.68 (8.16)	117.50 (11.39)	103.64 (12.23)^a,b,c,d^	7.83	<0.001	0.20
CEN	171.48 (17.42)	177.11 (16.40)	172.50 (12.59)	174.45 (17.34)	158.44 (18.63)^a,b,c,d^	4.36	0.002	0.12
SN	52.55 (5.53)	54.00 (5.30)	51.84 (3.68)	53.01 (5.66)	48.56 (3.12)^a,b,c,d^	4.17	0.003	0.12
CBCL – 12-months								
CBCL ADHD, *M* (s.d.)	51.47 (3.40)	53.19 (5.40)	54.64 (8.16)	55.35 (7.80)^e^	56.10 (6.45)^a^	2.59	0.040	0.08
CBCL AP, *M* (s.d.)	51.88 (2.80)	53.05 (4.36)	55.27 (9.79)	56.78 (10.04)^e^	57.10 (6.30)^a^	3.47	0.010	0.10

*Note*: Significant (*p* < 0.05) Bonferroni corrected post-hoc analyses comparing ^a^TD *v.* TBI-severe, ^b^TBI-mild *v.* TBI-severe, ^c^TBI-complicated-mild *v.* TBI-severe, ^d^TBI-moderate *v.* TBI-severe, ^e^TD *v.* TBI-moderate.

Group differences for the individual ROIs associated with each large-scale neural network are displayed in Table 4. As expected, post-hoc pairwise comparisons revealed that, for all individual ROIs within the DMN, CEN and SN, the severe TBI group showed significantly diminished volumes relative to TD controls (all *p* < 0.05), and children with mild, complicated-mild and moderate TBI (all *p* < 0.05) (see Table 4).

**Table 4.** Group differences in regions of interest associated with the DMN, CEN and SN

Network volume cm^3^ (s.d.)	TD control *n* = 34	Mild *n* = 53	Complicated-mild TBI *n* = 14	Moderate TBI *n* = 24	Severe TBI *n* = 12	*F*	*p*	*η*^2^
Default mode network								
Ventromedial prefrontal cortex	40.00 (4.39)	41.42 (3.83)	39.25 (2.19)^a^	40.80 (3.77)	34.91 (5.79)^b,c,d,e^	8.60	<0.001	0.21
Posterior cingulate cortex	17.38 (2.31)	17.71 (2.36)	16.55 (1.56)	17.33 (2.43)	15.35 (2.89)^b,c,d,e^	3.11	0.018	0.09
Inferior parietal lobule	50.20 (5.01)	50.54 (5.07)	50.26 (4.66)	50.41 (5.63)	45.66 (5.44)^b,c,d,e^	3.15	0.017	0.09
Hippocampal formation	8.92 (0.86)	9.02 (0.74)	8.61 (0.81)	8.97 (0.88)	7.72 (1.68)^b,c,d,e^	5.65	<0.001	0.15
Central executive network								
Dorsolateral prefrontal cortex	83.69 (10.48)	88.40 (10.15)	85.14 (7.23)	86.55 (9.61)	77.56 (11.31)^b,c,d,e^	3.88	0.005	0.11
Posterior parietal cortex	63.71 (6.22)	64.25 (6.11)	63.87 (5.68)	64.32 (7.74)	58.77 (7.26)^b,c,d,e^	2.46	0.049	0.07
Caudate Nucleus	8.09 (1.33)	8.25 (1.07)	7.98 (0.98)	7.78 (0.82)	7.46 (0.79)^c^	1.47	0.214	0.04
Thalamus	15.99 (1.47)	16.20 (1.46)	15.51 (1.23)	15.81 (1.33)	14.65 (1.99)^b,c,d,e^	3.54	0.009	0.10
Salience network								
Ventrolateral prefrontal cortex	3.73 (0.66)	3.83 (0.63)	3.81 (0.56)	3.85 (0.63)	3.26 (0.54)^b,c,d,e^	2.28	0.064	0.07
Insula	26.47 (2.92)	27.30 (2.92)	26.23 (2.01)	26.89 (3.01)	25.39 (1.75)^c^	1.25	0.293	0.04
Anterior cingulate cortex	19.09 (2.20)	19.48 (2.30)	18.73 (1.88)	19.02 (2.60)	16.98 (2.44)^b,c,d,e^	3.61	0.008	0.10
Amygdala	3.25 (0.44)	3.39 (0.40)	3.07 (0.34)	3.25 (0.46)	2.93 (0.47)^b,c,e^	3.10	0.018	0.09

*Note:* Significant (*p* < 0.05) Bonferroni post-hoc analyses comparing ^a^TBI-mild *v.* mild complex, ^b^TD *v.* TBI-severe, ^c^TBI-mild *v.* TBI-severe, ^d^TBI-mild complex *v.* TBI-severe, ^e^TBI-moderate *v.* TBI-severe.

### The impact of TBI on ADHD symptomatology

In total, 129 participants (43 TD, 42 mild TBI, 11 complicated mild TBI, 23 moderate TBI, 10 severe TBI) completed standardised parent-report measures of ADHD symptomatology at 12-months post-injury. A sensitivity analysis found no significant differences in the pre-injury, demographic or injury-related characteristics of those with and without 12-month ADHD symptom rating data (all *p* ⩾ 0.17).

#### TBI *v.* TD

As expected, the TBI group displayed significantly higher ADHD symptomatology than the TD group on the CBCL DSM-oriented ADHD Problems scale (*p* = 0.016; *η*^2^ = 0.045) and the CBCL Attention Problems scale (*p* = 0.043; *η*^2^ = 0.032). Similarly, we found that compared to the TD group, scores within the borderline-clinical range (*T* > 65) for the CBCL DSM-oriented ADHD Problems scale were significantly more common in the TBI group (TBI: 19% *v.* TD control: 2%, Fisher's exact *p* *=* 0.011). Although the TBI group also displayed higher rates of impairment than TD controls on the CBCL Attention Problems scale, the difference did not reach statistical significance (TBI: 14% *v.* TD control: 2%, Fisher's exact *p* = 0.059).

#### Effect of TBI severity group membership

Online Supplementary Figure S2 includes violin plots depicting s-ADHD symptom severity as a function of TBI severity group membership. As shown in Table 3, TBI severity group membership was significantly associated with scores on the CBCL DSM-oriented ADHD Problems scale (*p* = 0.04; *η*^2^ = 0.08) and the CBCL Attention Problems scale (*p* = 0.01; *η*^2^ = 0.10). Post-hoc tests showed that in comparison to TD children, children in the moderate TBI and severe TBI groups had significantly higher levels of inattention and DSM-oriented ADHD symptomology measured by the CBCL Attention Problems and ADHD Problem scales, respectively (see Table 3).

### Prospective associations between network-based brain morphometry and s-ADHD symptom severity

Multivariable regression models were used to evaluate the prognostic value of network-based brain morphometry for prospectively predicting s-ADHD symptom severity, after adjusting for age, sex, TIV and other established risk factors, including acute TBI severity, SES, family function and premorbid adaptive function. All variables had a VIF of less than 2.5, indicating a lack of multicollinearity between predictors. Bivariate correlations between ADHD symptom severity, network-based brain morphometry and other risk factor covariates are presented in online Supplementary Table S1.

The final adjusted regression model predicting CBCL DSM-oriented ADHD Problems was significant [*R*^2^ = 0.31, *F*_(9, 75)_ = 4.42, *p* < 0.001]. As shown in Table 5, DMN morphometry independently predicted ADHD symptomatology (*p* = 0.015), such that diminished DMN volume was independently associated with higher ADHD symptomatology. Greater ADHD symptomatology was also independently associated with poorer family functioning (*p* = 0.016) and lower pre-injury adaptive functioning (*p* = 0.005). The remaining covariates were not independently related to outcome (all *p* > 0.10).

**Table 5.** Multivariable regression models predicting ADHD symptom severity from network-based morphometry

	B	s.e. B	*t*	*p* value	95% CI
CBCL ADHD problems^a^					
DMN morphometry	−0.22	0.09	−2.49	0.015	(−0.39 to −0.43)
SN morphometry	−0.21	0.14	−1.53	0.132	(−0.06 to 0.48)
CEN morphometry	−0.09	0.06	−1.49	0.140	(−0.03 to 0.20)
CBCL attention problems^a^					
DMN morphometry	−0.21	0.10	−2.18	0.033	(−0.41 to −0.02)
SN morphometry	−0.23	0.18	−1.26	0.211	(−0.13 to 0.59)
CEN morphometry	−0.08	0.07	−1.18	0.244	(−0.06 to 0.21)

a Multivariable model adjusted for sex, estimated total intracranial volume, acute TBI severity, SES, pre-injury ABAS GAC, FAD general functioning.

The final adjusted regression model predicting CBCL Attention Problems was also significant [*R*^2^ = 0.30, *F*_(9,75)_ = 3.14, *p* = 0.003]. As shown in Table 5, DMN morphometry was an independent predictor of CBCL Attention Problems at 12-months post-injury (*p* = 0.033), such that diminished DMN volume was associated with higher attention problems. Family functioning was also an independent predictor of outcome (*p* = 0.025), such that worse family functioning was prospectively associated with more frequent attention problems. The remaining covariates were not independently related to outcome (all *p* > 0.10).

Moderation analyses using hierarchical regression models revealed no statistically significant interactions between brain network morphometry and history of surgical intervention on s-ADHD symptom severity in the TBI sample (DMN: *p* = 0.71; CEN: *p* = 0.47; SN: *p* = 0.39).

### Sensitivity analyses

To determine if findings were driven by children with severe TBI, multivariate regression models were re-run after excluding children with severe TBI. In keeping with the findings of the primary analyses reported above, all results remained highly similar when children with severe TBI were excluded (see online Supplementary Table S2).

## Discussion

Despite a well-established association of childhood TBI with elevated s-ADHD symptomatology, the neurostructural brain correlates of these post-injury symptoms remain largely unknown. To address this substantial gap in evidence, we aimed to assess the effect of childhood TBI on DMN, CEN and SN morphometry, and evaluate whether neurostructural alterations within these higher-order cognitive networks have independent prognostic value for predicting s-ADHD symptom severity. As expected, the severe TBI group showed altered network-based brain morphometry, including significantly diminished grey matter volume within the DMN, CEN and SN. In multivariable models adjusted for other well-established s-ADHD risk factors, altered DMN morphometry independently predicted higher s-ADHD symptomatology at 12-months post-injury, whilst SN and CEN morphometry were not significant independent predictors. Findings from our relatively large childhood TBI cohort without preinjury ADHD suggest that neurostructural alterations within higher-order cognitive circuitry may represent a prospective risk factor for elevated s-ADHD symptomatology at 12-months post-injury.

Compared to the TD group, rates of clinically elevated ADHD symptomatology were significantly greater in the TBI sample overall. Similarly, when symptoms were evaluated dimensionally, children with TBI had significantly higher ADHD symptomatology than their TD peers 12-months post-injury. Although the association of childhood TBI with elevated ADHD symptomatology is relatively well documented (Bellesi, Barker, Brown, & Valmaggia, 2019; Li & Liu, 2013), our findings are the first to report this relationship in a sample of Australian children with medically confirmed TBI and no pre-injury ADHD. Since elevated s-ADHD symptomatology appears evident at 12-months post-injury, our findings emphasise the value of routine follow-up and where clinically indicated, early preventive measures for those children exhibiting risk factors for s-ADHD symptomatology, e.g. brain structural abnormalities, lower pre-injury adaptive function and poorer post-injury family function.

Consistent with prior research (Asarnow et al., 2021; Narad et al., 2018), we found some evidence of a dose–response association between acute TBI severity and s-ADHD symptomatology. While the mild TBI group did not significantly differ from the TD group, children with moderate and severe TBI showed significantly higher ADHD symptomatology than their age-and-sex matched peers. Despite some evidence of a dose–response relationship, the group means of *all* TBI severity groups fell within normative limits and were notable for their large standard deviations. These findings suggest that acute TBI severity does not fully account for the substantial individual heterogeneity in s-ADHD symptomatology (Asarnow et al., 2021), particularly among children with more severe TBI. Consequently, the current findings reinforce the importance of theory-driven approaches to prediction of s-ADHD symptomology, including the need to identify neurostructural risk factors linking childhood TBI to elevated ADHD symptomatology.

Guided by the influential TN theory of ADHD (Menon, 2011), we sought to assess the effect of childhood TBI on brain morphometry of higher-order cognitive networks proposed to play a key role in ADHD pathophysiology (Cai et al., 2018; Castellanos & Proal, 2012). In line with previous reports of grey matter neurostructural alterations following childhood TBI (Dennis et al., 2013), children with severe TBI showed altered brain morphometry within large-scale cognitive networks, including significantly diminished grey matter volume within the DMN, SN and CEN. Interestingly, altered regional brain volumes within these large-scale neural networks was documented alongside relative preservation of global brain volumes (e.g. total white matter, TIV), which did not significantly differ between groups. Although these findings suggest that large-scale, higher-order cognitive networks may be selectively vulnerable to the sub-acute effects of severe childhood TBI, to our knowledge no study has evaluated whether DMN, CEN and SN morphometry have independent prognostic value for predicting s-ADHD symptom severity in children with TBI.

In modelling these theory-driven relationships between network-based brain morphometry and s-ADHD symptom severity, we adjusted for other well-established s-ADHD risk factors, including acute TBI severity, post-injury family function, family SES and estimates of pre-injury child adaptive skills. Of these covariates in the final adjusted models, we found that lower pre-injury adaptive function and poorer post-injury family function were independently associated with higher s-ADHD symptomatology. Interestingly, acute TBI severity did not independently contribute to s-ADHD symptomatology in the final adjusted model. This finding is consistent with prior research by Max and et al. (1998), who found that the effect of TBI severity on s-ADHD symptom severity was not statistically significant after adjusting for pre-injury and family environmental factors. Though the current findings are broadly consistent with prior research suggesting that pre-injury and family-based risk factors may interact with injury-related characteristics to heighten vulnerability to chronic neurobehavioral sequelae (Gerring & Wade, 2012), our primary aim was to evaluate if neurostructural alterations within higher-order cognitive circuitry represent an independent, prospective risk factor for s-ADHD symptomatology in children with TBI.

In partial support of predictions, altered DMN morphometry independently predicted higher s-ADHD symptomatology at 12-months post-injury, whilst SN and CEN morphometry did not. Although the mechanism underlying this relation remains to be established, the TN model of ADHD may offer a useful framework to interpret the current findings. For example, extensive evidence suggests that top-down cognitive processes rely on the coordinated activity of multiple large-scale brain networks that are fundamentally altered in ADHD (Cai et al., 2016, 2018; Chen et al., 2015; Sridharan et al., 2008; Supekar & Menon, 2012). Specifically, numerous reports show that top-down cognitive processes are dependent on the diminishing activation of the DMN in synchrony with activation of task-relevant networks (Baum et al., 2017; Gu et al., 2015; Satterthwaite et al., 2013), including the SN, which modulates de-activation of the DMN during top-down cognitive processing (Crockett, Hsu, Best, & Liu-Ambrose, 2017). Given that diminished DMN volume may represent a proxy for disruption to intra- and inter-network structural connectivity (Bigler, 2013; Spitz et al., 2013), s-ADHD symptoms after childhood TBI may be at least partly related to structural disconnection of the DMN from task-relevant networks, which is likely to constrain functional connectivity (Johnston et al., 2008). Such constraints may contribute to failure of the DMN to deactivate in synchrony with activation of task-relevant, cognitive control networks (Dennis et al., 2013; Satterthwaite et al., 2013).

An alternative account suggests that the DMN plays a critical role in making memory-based predictions to support decision-making under established behavioural contexts (Vatansever, Menon, & Stamatakis, 2017). Based on this account, one might predict that structural damage to the DMN may contribute to difficulties in maintaining detailed task representations, manifesting as ADHD-like behaviours (Vatansever, Bozhilova, Asherson, & Smallwood, 2019). Taken together with previous reports suggesting that higher-order cognitive networks including the DMN are fundamentally altered in children with ADHD (Cai et al., 2018), our finding that altered DMN morphometry is a prospective risk factor for s-ADHD symptomology in children with TBI is perhaps not surprising.

Although the DMN was robustly associated with ADHD symptom severity, the CEN and SN did not independently contribute to s-ADHD symptom burden in our TBI sample. The non-significant effect of the SN and CEN on s-ADHD symptom severity may be partly explained by the limited scope of our longitudinal follow-up over time post-injury. Namely, we assessed s-ADHD symptom severity at 12-months post-injury when the developmental consequences of TBI may not be fully realised in our relatively young sample of children for whom higher-order cognitive networks are still undergoing rapid structural and functional maturation (Sherman et al., 2014). Given the protracted maturational timetable of the CEN and SN (Satterthwaite et al., 2013; Sherman et al., 2014), it is possible that the behavioural consequences of damage to these neural networks does not become apparent until later during development, i.e. beyond 12-month post-injury. Accordingly, further longitudinal follow-up is required to evaluate whether the hypothesised pattern of relationships between the CEN, SN and s-ADHD symptom severity becomes evident beyond 12-months post-injury.

### Implications

Our findings address the dearth of well-powered, theory-driven research to identify neurostructural brain correlates of elevated s-ADHD symptomatology in children with TBI. Specifically, our study provides the first evidence for a robust prospective association between altered DMN morphometry and s-ADHD symptomatology in children with TBI and no preinjury ADHD. These findings suggest that when used to complement routine screening for other established risk factors, high-resolution structural brain MRI has potential to unlock early prognostic biomarkers, which may aid early identification of high-risk children with TBI who could benefit from ongoing surveillance and/or clinically indicated interventions to address modifiable risk factors.

From a broader clinical perspective, our findings suggest that elevated s-ADHD symptomatology is linked to several independent, prospective risk factors, including worse premorbid function, poorer post-injury family function and neurostructural alterations in higher-order cognitive circuitry. These findings suggest that clinicians should exercise caution when prognosticating post-injury outcomes based on acute TBI severity alone and underscore the need to consider the potential interplay of multiple biopsychosocial factors contributing to a child's post-injury neuropsychiatric presentation. Encouragingly, the prospective association between post-injury family functioning and ADHD-symptom severity underscores the potential value of family-based interventions that address potentially modifiable risk factors for s-ADHD symptomatology (Kurowski et al., 2020).

### Limitations and future directions

The current study is limited by a sole reliance on standardised parent ratings of s-ADHD symptoms, which cannot be used to establish rates of formal DSM-ADHD diagnoses and may underestimate the frequency and severity of symptoms in other contexts (e.g. school environment). To address this caveat and strengthen the quality of evidence regarding the prevalence of s-ADHD and its correlates after childhood TBI, future research should incorporate structured clinical interviews and standardised teacher ratings of ADHD symptoms in larger samples of children with more severe TBI. Furthermore, while the current study is strengthened by the inclusion of a well-matched group of TD children, future research should incorporate an additional orthopaedic injury comparison group to establish whether the current pattern of findings is brain injury-specific.

Secondly, we cannot entirely rule out the possibility that s-ADHD symptoms in our TBI sample are at least partly explained by pre-injury psychiatric and/or developmental vulnerabilities, including symptoms of ADHD and disruptive behaviour, which may place children at elevated risk for TBI. Nevertheless, there are several factors that do not support this possibility. Firstly, retrospective ratings of pre-injury functioning showed that the TBI groups did not significantly differ from TD controls on measures of pre-injury externalizing symptoms, adaptive behaviour or internalizing symptoms. Viewed collectively with the finding that all results remained statistically significant after adjusting for pre-injury externalizing symptoms, our results suggest that s-ADHD symptoms in our sample are more likely explained by brain injury-related processes than neurodevelopmental vulnerabilities pre-dating the injury.

Moreover, because we relied on structural imaging data collected at a single, sub-acute time-point to evaluate prospective associations between network-based brain morphometry and s-ADHD symptom severity, causal relationships between these variables cannot be established, and the stability of the structural brain abnormalities in the long-term post-injury could not be assessed in this study. Additionally, since structural brain MRI was acquired during the acute/sub-acute recovery period when valid and reliable estimates of s-ADHD symptom severity could not be obtained concurrently with the structural MRI, we were unable to examine cross-sectional associations between sub-acute volumetric differences and concurrent s-ADHD symptom severity.

Finally, our imaging protocol used only structural imaging techniques and did not include any functional neuroimaging sequences (e.g. resting state connectivity) that may have provided additional information regarding the neural correlates of s-ADHD symptoms in our sample. To address this limitation, future research using functional MRI would help to elucidate whether s-ADHD symptom severity is related to aberrancies in time-varying engagement of the SN with the DMN and CEN in children with TBI.

## Conclusions

In addressing the paucity of prospective research to identify neurostructural brain correlates of elevated s-ADHD symptomology after childhood TBI, we show that childhood TBI is associated with significantly higher s-ADHD symptomatology and selective neurostructural alterations within higher-order cognitive networks proposed to play a key role in ADHD pathophysiology. After adjusting for other well-established risk factors, we found that altered DMN morphometry was prospectively associated with higher s-ADHD symptomatology at 12-months post-injury. These findings suggest that neurostructural alterations within higher-order cognitive circuitry may represent a potential prognostic marker of s-ADHD symptomatology, which may aid early identification of high-risk children with TBI who could benefit from early surveillance and preventive measures to optimise long-term neuropsychiatric outcomes.

## Supplementary material

For supplementary material accompanying this paper visit https://doi.org/10.1017/S0033291722002598. click here to view supplementary material

## References

[ref1] Achenbach, T., & Rescorla, L. (2001). Manual for ASEBA school-age forms and profiles. Burlington, VT: Research Center for Children Youth and Families, University of Vermont.

[ref2] American Psychiatric Association. (2013). Diagnostic and statistical manual of mental disorders (5th ed.). Arlington, VA: American Psychiatric Association.

[ref3] Anderson, V., Beauchamp, M. H., Yeates, K. O., Crossley, L., Ryan, N. P., Hearps, S. J., & Catroppa, C. (2017). Social competence at 2 years following child traumatic brain injury. Journal of Neurotrauma, 34(14), 2261–2271. 28177265 10.1089/neu.2016.4692

[ref4] Asarnow, R. F., Newman, N., Weiss, R. E., & Su, E. (2021). Association of attention-deficit/hyperactivity disorder diagnoses with pediatric traumatic brain injury: A meta-analysis. JAMA Pediatrics, 175(10), 1009–1016. 34251435 10.1001/jamapediatrics.2021.2033 PMC8276124

[ref5] Baum, G. L., Ciric, R., Roalf, D. R., Betzel, R. F., Moore, T. M., Shinohara, R. T., … Quarmley, M. (2017). Modular segregation of structural brain networks supports the development of executive function in youth. Current Biology, 27(11), 1561–1572. 28552358 10.1016/j.cub.2017.04.051 PMC5491213

[ref6] Bellesi, G., Barker, E. D., Brown, L., & Valmaggia, L. (2019). Pediatric traumatic brain injury and antisocial behavior: Are they linked? A systematic review. Brain Injury, 33(10), 1272–1292. 31327257 10.1080/02699052.2019.1641621

[ref7] Bigler, E. D. (2013). Traumatic brain injury, neuroimaging, and neurodegeneration. Frontiers in Human Neuroscience, 7, 395–405. 23964217 10.3389/fnhum.2013.00395 PMC3734373

[ref8] Cai, W., Chen, T., Ryali, S., Kochalka, J., Li, C.-S. R., & Menon, V. (2016). Causal interactions within a frontal-cingulate-parietal network during cognitive control: Convergent evidence from a multisite–multitask investigation. Cerebral Cortex, 26(5), 2140–2153. 25778346 10.1093/cercor/bhv046 PMC4830290

[ref9] Cai, W., Chen, T., Szegletes, L., Supekar, K., & Menon, V. (2018). Aberrant time-varying cross-network interactions in children with attention-deficit/hyperactivity disorder and the relation to attention deficits. Biological Psychiatry: Cognitive Neuroscience and Neuroimaging, 3(3), 263–273. 29486868 10.1016/j.bpsc.2017.10.005 PMC5833018

[ref10] Cai, W., Ryali, S., Chen, T., Li, C.-S. R., & Menon, V. (2014). Dissociable roles of right inferior frontal cortex and anterior insula in inhibitory control: Evidence from intrinsic and task-related functional parcellation, connectivity, and response profile analyses across multiple datasets. Journal of Neuroscience, 34(44), 14652–14667. 25355218 10.1523/JNEUROSCI.3048-14.2014 PMC4212065

[ref11] Castellanos, F. X., & Proal, E. (2012). Large-scale brain systems in ADHD: Beyond the prefrontal–striatal model. Trends in Cognitive Sciences, 16(1), 17–26. 22169776 10.1016/j.tics.2011.11.007 PMC3272832

[ref12] Chen, T., Michels, L., Supekar, K., Kochalka, J., Ryali, S., & Menon, V. (2015). Role of the anterior insular cortex in integrative causal signaling during multisensory auditory–visual attention. European Journal of Neuroscience, 41(2), 264–274. 25352218 10.1111/ejn.12764 PMC4300257

[ref13] Cortese, S., Kelly, C., Chabernaud, C., Proal, E., Di Martino, A., Milham, M. P., & Castellanos, F. X. (2012). Toward systems neuroscience of ADHD: A meta-analysis of 55 fMRI studies. American Journal of Psychiatry, 169(10), 1038–1055. 22983386 10.1176/appi.ajp.2012.11101521 PMC3879048

[ref14] Crockett, R. A., Hsu, C. L., Best, J. R., & Liu-Ambrose, T. (2017). Resting state default mode network connectivity, dual task performance, gait speed, and postural sway in older adults with mild cognitive impairment. Frontiers in Aging Neuroscience, 9, 423–430. 29311906 10.3389/fnagi.2017.00423 PMC5742581

[ref15] Dennis, M., Simic, N., Bigler, E. D., Abildskov, T., Agostino, A., Taylor, H. G., … Stancin, T. (2013). Cognitive, affective, and conative theory of mind (ToM) in children with traumatic brain injury. Developmental Cognitive Neuroscience, 5, 25–39. 23291312 10.1016/j.dcn.2012.11.006 PMC3620837

[ref16] Desikan, R. S., Ségonne, F., Fischl, B., Quinn, B. T., Dickerson, B. C., Blacker, D., … Hyman, B. T. (2006). An automated labeling system for subdividing the human cerebral cortex on MRI scans into gyral based regions of interest. Neuroimage, 31(3), 968–980. 16530430 10.1016/j.neuroimage.2006.01.021

[ref17] Dewan, M. C., Mummareddy, N., Wellons, III, J. C., & Bonfield, C. M. (2016). Epidemiology of global pediatric traumatic brain injury: Qualitative review. World Neurosurgery, 91, 497–509. 27018009 10.1016/j.wneu.2016.03.045

[ref18] Emery, C. A., Barlow, K. M., Brooks, B. L., Max, J. E., Villavicencio-Requis, A., Gnanakumar, V., … Yeates, K. O. (2016). A systematic review of psychiatric, psychological, and behavioural outcomes following mild traumatic brain injury in children and adolescents. The Canadian Journal of Psychiatry, 61(5), 259–269. 27254800 10.1177/0706743716643741 PMC4841286

[ref19] Epstein, N. B., Baldwin, L. M., & Bishop, D. S. (1983). The McMaster family assessment device. Journal of Marital Family Therapy, 9(2), 171–180.

[ref20] Fischl, B., & Dale, A. M. (2000). Measuring the thickness of the human cerebral cortex from magnetic resonance images. Proceedings of the National Academy of Sciences, 97(20), 11050–11055. 10.1073/pnas.200033797 PMC27146 10984517

[ref21] Fischl, B., Salat, D. H., Busa, E., Albert, M., Dieterich, M., Haselgrove, C., … Klaveness, S. (2002). Whole brain segmentation: Automated labeling of neuroanatomical structures in the human brain. Neuron, 33(3), 341–355. 11832223 10.1016/s0896-6273(02)00569-x

[ref22] Fischl, B., Salat, D. H., Van Der Kouwe, A. J., Makris, N., Ségonne, F., Quinn, B. T., & Dale, A. M. (2004). Sequence-independent segmentation of magnetic resonance images. Neuroimage, 23, S69–S84. 15501102 10.1016/j.neuroimage.2004.07.016

[ref23] Fischl, B., Sereno, M. I., Tootell, R. B., & Dale, A. M. (1999). High-resolution intersubject averaging and a coordinate system for the cortical surface. Human Brain Mapping, 8(4), 272–284. 10619420 10.1002/(SICI)1097-0193(1999)8:4<272::AID-HBM10>3.0.CO;2-4 PMC6873338

[ref24] Francx, W., Llera, A., Mennes, M., Zwiers, M. P., Faraone, S. V., Oosterlaan, J., … Franke, B. (2016). Integrated analysis of gray and white matter alterations in attention-deficit/hyperactivity disorder. NeuroImage: Clinical, 11, 357–367. 27298764 10.1016/j.nicl.2016.03.005 PMC4893015

[ref25] Gerring, J. P., Brady, K. D., Chen, A., Vasa, R., Grados, M., Bandeen-Roche, K. J., … Denckla, M. B. (1998). Premorbid prevalence of ADHD and development of secondary ADHD after closed head injury. Journal of the American Academy of Child and Adolescent Psychiatry, 37(6), 647–654. 9628085 10.1097/00004583-199806000-00015

[ref26] Gerring, J. P., & Wade, S. (2012). The essential role of psychosocial risk and protective factors in pediatric traumatic brain injury research. Journal of Neurotrauma, 29(4), 621–628. 22091875 10.1089/neu.2011.2234 PMC3289845

[ref27] Gu, S., Satterthwaite, T. D., Medaglia, J. D., Yang, M., Gur, R. E., Gur, R. C., & Bassett, D. S. (2015). Emergence of system roles in normative neurodevelopment. Proceedings of the National Academy of Sciences, 112(44), 13681–13686. 10.1073/pnas.1502829112 PMC4640772 26483477

[ref28] Gusnard, D. A., Akbudak, E., Shulman, G. L., & Raichle, M. E. (2001). Medial prefrontal cortex and self-referential mental activity: Relation to a default mode of brain function. Proceedings of the National Academy of Sciences, 98(7), 4259–4264. 10.1073/pnas.071043098 PMC31213 11259662

[ref29] Harrison, P., & Oakland, T. (2003). Adaptive behavior assessment system (ABAS-II). San Antonio, TX: The Psychological Corporation.

[ref30] Hoskinson, K. R., Bigler, E. D., Abildskov, T. J., Dennis, M., Taylor, H. G., Rubin, K., … Yeates, K. O. (2019). The mentalizing network and theory of mind mediate adjustment after childhood traumatic brain injury. Social Cognitive and Affective Neuroscience, 14(12), 1285–1295. 31993655 10.1093/scan/nsaa006 PMC7137721

[ref31] Johnston, J. M., Vaishnavi, S. N., Smyth, M. D., Zhang, D., He, B. J., Zempel, J. M., … Raichle, M. E. (2008). Loss of resting interhemispheric functional connectivity after complete section of the corpus callosum. The Journal of Neuroscience 28(25), 6453–6458. 18562616 10.1523/JNEUROSCI.0573-08.2008 PMC2738991

[ref32] Jovicich, J., Czanner, S., Han, X., Salat, D., van der Kouwe, A., Quinn, B., … Blacker, D. (2009). MRI-derived measurements of human subcortical, ventricular and intracranial brain volumes: Reliability effects of scan sessions, acquisition sequences, data analyses, scanner upgrade, scanner vendors and field strengths. Neuroimage, 46(1), 177–192. 19233293 10.1016/j.neuroimage.2009.02.010 PMC2866077

[ref33] Kurowski, B. G., Taylor, H. G., McNally, K. A., Kirkwood, M. W., Cassedy, A., Horn, P. S., … Wade, S. L. (2020). Online family problem-solving therapy (F-PST) for executive and behavioral dysfunction after traumatic brain injury in adolescents: A randomized, multicenter, comparative effectiveness clinical trial. The Journal of Head Trauma Rehabilitation, 35(3), 165–174. 31834062 10.1097/HTR.0000000000000545 PMC7205575

[ref34] Li, L., & Liu, J. (2013). The effect of pediatric traumatic brain injury on behavioral outcomes: A systematic review. Developmental Medicine and Child Neurology, 55(1), 37–45. 22998525 10.1111/j.1469-8749.2012.04414.x PMC3593091

[ref35] Max, J. E., Arndt, S., Castillo, C. S., Bokura, H., Robin, D. A., Lindgren, S. D., … Mattheis, P. J. (1998). Attention-deficit hyperactivity symptomatology after traumatic brain injury: A prospective study. Journal of the American Academy of Child and Adolescent Psychiatry, 37(8), 841–847. 9695446 10.1097/00004583-199808000-00014

[ref36] Max, J. E., Schachar, R. J., Levin, H. S., Ewing-Cobbs, L., Chapman, S. B., Dennis, M., … Landis, J. (2005a). Predictors of attention-deficit/hyperactivity disorder within 6 months after pediatric traumatic brain injury. Journal of the American Academy of Child and Adolescent Psychiatry, 44(10), 1032–1040. 16175108 10.1097/01.chi.0000173293.05817.b1

[ref37] Max, J. E., Schachar, R. J., Levin, H. S., Ewing-Cobbs, L., Chapman, S. B., Dennis, M., … Landis, J. (2005b). Predictors of secondary attention-deficit/hyperactivity disorder in children and adolescents 6 to 24 months after traumatic brain injury. Journal of the American Academy of Child and Adolescent Psychiatry, 44(10), 1041–1049. 16175109 10.1097/01.chi.0000173292.05817.f8

[ref38] Max, J. E., Wilde, E. A., Bigler, E. D., MacLeod, M., Vasquez, A. C., Schmidt, A. T., … Levin, H. S. (2012). Psychiatric disorders after pediatric traumatic brain injury: A prospective, longitudinal, controlled study. The Journal of Neuropsychiatry and Clinical Neurosciences, 24(4), 427–436. 23224448 10.1176/appi.neuropsych.12060149

[ref39] McMillan, J., Beavis, A., & Jones, F. L. (2009). The AUSEI06 a new socioeconomic index for Australia. Journal of Sociology, 45(2), 123–149.

[ref40] Menon, V. (2011). Large-scale brain networks and psychopathology: A unifying triple network model. Trends in Cognitive Sciences, 15(10), 483–506. 21908230 10.1016/j.tics.2011.08.003

[ref41] Narad, M. E., Kennelly, M., Zhang, N., Wade, S. L., Yeates, K. O., Taylor, H. G., … Kurowski, B. G. (2018). Secondary attention-deficit/hyperactivity disorder in children and adolescents 5 to 10 years after traumatic brain injury. JAMA Pediatrics, 172(5), 437–443. 29554197 10.1001/jamapediatrics.2017.5746 PMC5875309

[ref42] Owen, A. M., McMillan, K. M., Laird, A. R., & Bullmore, E. (2005). N-back working memory paradigm: A meta-analysis of normative functional neuroimaging studies. Human Brain Mapping, 25(1), 46–59. 15846822 10.1002/hbm.20131 PMC6871745

[ref43] Risen, S., Barber, A. D., Mostofsky, S., & Suskauer, S. (2015). Altered functional connectivity in children with mild to moderate TBI relates to motor control. Journal of Pediatric Rehabilitation Medicine, 8(4), 309–319. 26684071 10.3233/PRM-150349 PMC4861163

[ref44] Robinson, K. E., Fountain-Zaragoza, S., Dennis, M., Taylor, H. G., Bigler, E. D., Rubin, K., … Yeates, K. O. (2014). Executive functions and theory of mind as predictors of social adjustment in childhood traumatic brain injury. Journal of Neurotrauma, 31(22), 1835–1842. 25003478 10.1089/neu.2014.3422 PMC4224037

[ref45] Ryan, N. P., Catroppa, C., Hughes, N., Painter, F. L., Hearps, S., Beauchamp, M. H., & Anderson, V. A. (2021). Executive function mediates the prospective association between neurostructural differences within the central executive network and anti-social behavior after childhood traumatic brain injury. The Journal of Child Psychology and Psychiatry 62(9), 1150–1161. 33624844 10.1111/jcpp.13385

[ref46] Satterthwaite, T. D., Wolf, D. H., Erus, G., Ruparel, K., Elliott, M. A., Gennatas, E. D., … Bilker, W. B. (2013). Functional maturation of the executive system during adolescence. Journal of Neuroscience, 33(41), 16249–16261. 24107956 10.1523/JNEUROSCI.2345-13.2013 PMC3792462

[ref47] Ségonne, F., Pacheco, J., & Fischl, B. (2007). Geometrically accurate topology-correction of cortical surfaces using nonseparating loops. IEEE Transactions on Medical Imaging, 26(4), 518–529. 17427739 10.1109/TMI.2006.887364

[ref48] Sherman, L. E., Rudie, J. D., Pfeifer, J. H., Masten, C. L., McNealy, K., & Dapretto, M. (2014). Development of the default mode and central executive networks across early adolescence: A longitudinal study. Developmental Cognitive Neuroscience, 10, 148–159. 25282602 10.1016/j.dcn.2014.08.002 PMC4854607

[ref49] Sled, J. G., Zijdenbos, A. P., & Evans, A. C. (1998). A nonparametric method for automatic correction of intensity nonuniformity in MRI data. IEEE Transactions on Medical Imaging, 17(1), 87–97. 9617910 10.1109/42.668698

[ref50] Spitz, G., Bigler, E. D., Abildskov, T., Maller, J. J., O'Sullivan, R., & Ponsford, J. L. (2013). Regional cortical volume and cognitive functioning following traumatic brain injury. Brain and Cognition, 83(1), 34–44. 23872098 10.1016/j.bandc.2013.06.007

[ref51] Sridharan, D., Levitin, D. J., & Menon, V. (2008). A critical role for the right fronto-insular cortex in switching between central-executive and default-mode networks. Proceedings of the National Academy of Sciences, 105(34), 12569–12574. 10.1073/pnas.0800005105 PMC2527952 18723676

[ref52] Stephens, J. A., Salorio, C. F., Barber, A. D., Risen, S. R., Mostofsky, S. H., & Suskauer, S. J. (2018). Preliminary findings of altered functional connectivity of the default mode network linked to functional outcomes one year after pediatric traumatic brain injury. Developmental Neurorehabilitation, 21(7), 423–430. 28692408 10.1080/17518423.2017.1338777 PMC5843556

[ref53] Su, S., Chen, Y., Dai, Y., Lin, L., Qian, L., Zhou, Q., … Xiang, X. (2021). Quantitative synthetic MRI reveals grey matter abnormalities in children with drug-naïve attention-deficit/hyperactivity disorder. Brain Imaging and Behavior, 16, 406–414. doi:10.1007/s11682-021-00514-8. 34491528

[ref54] Supekar, K., & Menon, V. (2012). Developmental maturation of dynamic causal control signals in higher-order cognition: A neurocognitive network model. PLoS Computational Biology, 8(2), e1002374. 22319436 10.1371/journal.pcbi.1002374 PMC3271018

[ref55] Vatansever, D., Bozhilova, N. S., Asherson, P., & Smallwood, J. (2019). The devil is in the detail: Exploring the intrinsic neural mechanisms that link attention-deficit/hyperactivity disorder symptomatology to ongoing cognition. Psychological Medicine, 49(7), 1185–1194. 30514410 10.1017/S0033291718003598

[ref56] Vatansever, D., Menon, D. K., & Stamatakis, E. (2017). Default mode contributions to automated information processing. Proceedings of the National Academy of Sciences, 114(48), 12821–12826. 10.1073/pnas.1710521114 PMC5715758 29078345

[ref57] Wager, T. D., & Smith, E. E. (2003). Neuroimaging studies of working memory: A meta-analysis. Cognitive, Affective, & Behavioral Neuroscience, 3(4), 255–274. 10.3758/cabn.3.4.255 15040547

