# Supplementary material for: Association of neurostructural biomarkers with secondary attention-deficit/hyperactivity disorder (ADHD) symptom severity in children with traumatic brain injury: a prospective cohort study
Source: Psychol Med. 2022 Aug 25;53(11):5291–300. doi: 10.1017/S0033291722002598 (PMC10476057; doi:10.1017/S0033291722002598)
Supplement: Supplementary file 1 [file S0033291722002598sup001.zip › S0033291722002598sup002.docx]

*Fig. S2. Violin plots depicting s-ADHD symptom severity as a function of TBI severity group membership.*
