# Supplementary material for: Association of neurostructural biomarkers with secondary attention-deficit/hyperactivity disorder (ADHD) symptom severity in children with traumatic brain injury: a prospective cohort study
Source: Psychol Med. 2022 Aug 25;53(11):5291–300. doi: 10.1017/S0033291722002598 (PMC10476057; doi:10.1017/S0033291722002598)
Supplement: Supplementary file 1 [file S0033291722002598sup001.zip › S0033291722002598sup005.docx]

| **Table S1: Prospective associations between network-based morphometry, risk factor covariates, and ADHD symptom severity, *r* (*p*)** | | | | | | |
| --- | --- | --- | --- | --- | --- | --- |
|  | TBI group | | | | Typically developing group | |
|  | CBCL ADHD Problems | | CBCL Attention Problems | | CBCL ADHD Problems | CBCL Attention Problems |
| DMN morphometry | -.282 (.014) | | -.246 (.032) | | -.147 (.406) | .029 (.872) |
| SN morphometry | -.120 (.301) | | -.141 (.226) | | -.088 (.623) | .050 (.779) |
| CEN morphometry | -.150 (.196) | | -.179 (.122) | | -.057 (.751) | .110 (.537) |
| Acute TBI severity  Socio-economic status  Pre-injury adaptive behavior  Family functioning | -.180 (.115)  .093 (.421)  -.336 (.003)  .302 (.006) | | -.272 (.016)  .046 (.688)  -.303 (.007)  .286 (.009) | | -  -.359 (.018)  -  .102 (.514) | -  -.369 (.015)  -  .154 (.323) |
| Sex | .014 (.903) | | -.015 (.895) | | .023 (.885) | -.150 (.338) |
| Age | -.036 (.752) | | .018 (.873) | | .212 (.172) | .236 (.127) |
|  | | | | | |  |
|  |  |  | |  | |  |
