# Supplementary material for: Association of neurostructural biomarkers with secondary attention-deficit/hyperactivity disorder (ADHD) symptom severity in children with traumatic brain injury: a prospective cohort study
Source: Psychol Med. 2022 Aug 25;53(11):5291–300. doi: 10.1017/S0033291722002598 (PMC10476057; doi:10.1017/S0033291722002598)
Supplement: Supplementary file 1 [file S0033291722002598sup001.zip › S0033291722002598sup006.docx]

**Table S2. Regression models predicting ADHD symptom severity in children with mild-moderate TBI only**

|  | *B* | *SE B* | *t* | *p* value | 95% CI |
| --- | --- | --- | --- | --- | --- |
| **CBCL ADHD Problems*** |  |  |  |  |  |
| DMN morphometry | -.253 | .099 | -2.56 | .013 | [-.451, -.055] |
| SN morphometry | .209 | .139 | 1.50 | .139 | [-.070, .487] |
| CEN morphometry | .108 | .068 | 1.58 | .119 | [-.028, .244] |
| **CBCL Attention Problems*** |  |  |  |  |  |
| DMN morphometry | -.215 | .105 | -2.05 | .045 | [-.426, -.005] |
| SN morphometry | .214 | .145 | 1.47 | .146 | [-.077, .505] |
| CEN morphometry | .097 | .073 | 1.32 | .191 | [-.050, .243] |

*Multivariable model adjusted for sex, estimated total intracranial volume, acute TBI severity, SES, pre-injury

ABAS GAC, FAD General Functioning
